# Beyond the Image: Pitfalls, Cognitive Biases, and Safety Gaps in Non-Cardiac Neonatal Point-of-Care Ultrasound

**DOI:** 10.3390/children13101352

**Published:** 2026-10-05

**Authors:** Adrian Costache, Diana Iulia Vasilescu, Sorin Liviu Vasilescu, Andreea Raluca Gogoncea, Adriana Mihaela Dan

**Affiliations:** 1 Faculty of Medicine, “Carol Davila” University of Medicine and Pharmacy, 020956 Bucharest, Romania; costache.adi@gmail.com (A.C.); sorin-liviu.vasilescu@umfcd.ro (S.L.V.); adriana.dan@umfcd.ro (A.M.D.); 2 Department of Neonatology, Emergency University Hospital Bucharest, 050098 Bucharest, Romania; 3 Department of Obstetrics and Gynecology, Emergency University Hospital Bucharest, 050098 Bucharest, Romania; 4 Department of Neonatology, “Grigore Alexandrescu” Clinical Emergency Children Hospital Bucharest, 011743 Bucharest, Romania; dr.andreea.gogoncea@gmail.com

**Keywords:** point-of-care ultrasound, neonatology, lung ultrasound, cranial ultrasound, patient safety, cognitive bias, diagnostic error, ALARA, infection control, quality assurance

## Abstract

**Highlights:**

**What are the main findings?**

- Errors in non-cardiac neonatal POCUS can be grouped into four domains (indication, acquisition, interpretation, and clinical integration), and the most harmful often follow a technically adequate scan.
- Look-alike findings, bedside cognitive bias, and gaps in probe hygiene and output control are recurring and largely preventable sources of harm.

**What are the implications of the main findings?**

- Units that use POCUS need a stepwise, competency-based pathway for training, supervision, credentialing, image archiving, and quality review.
- Structured reporting, simple debiasing habits, and routine collaboration with radiology and cardiology can make neonatal POCUS safer without slowing bedside care.

**Abstract:**

Point-of-care ultrasound (POCUS) is now part of everyday work in neonatal intensive care, supporting diagnosis, procedures, and decisions in acutely unwell infants. Its spread has outpaced the development of training, credentialing, and quality assurance structures, and the neonatal literature rarely describes how and why the technique fails. In this perspective, we focus on non-cardiac neonatal POCUS (lung, cranial, and abdominal). We adapt existing frameworks into four error domains: indication, image acquisition, image interpretation, and clinical integration. We also separate errors that can occur in any ultrasound examination from those tied to the point-of-care setting, including confirmation, anchoring, and availability bias, time pressure, and fatigue. Using illustrative cases and comparative tables, we describe common look-alike findings, summarize thermal and mechanical bioeffects by organ, and discuss probe and gel hygiene in the neonatal unit. We then outline a stepwise pathway for training, supervision, credentialing, and quality assurance, and suggest practical safeguards: structured protocols and reports, simple debiasing habits, image archiving with periodic expert review, and close collaboration with radiology and cardiology. Seen in this way, investment in safety is part of the value of neonatal POCUS rather than an added cost.

## 1. Introduction

Point-of-care ultrasound (POCUS) is an examination performed and interpreted at the bedside by the clinician caring for the patient to answer a focused diagnostic, therapeutic, or procedural question [1]. After decades of use in adult emergency and critical care, it has become a routine tool in the neonatal intensive care unit (NICU). Current neonatal applications include lung, cranial, abdominal, urogenital, and vascular imaging, as well as guidance for vascular access, lumbar puncture, thoracentesis, and paracentesis and confirmation of endotracheal tube position [2]. Lung ultrasound remains the most thoroughly validated diagnostic application [2,3].

Guidance has followed this growth. The POCUS Working Group of the European Society of Paediatric and Neonatal Intensive Care (ESPNIC) issued international evidence-based guidelines in 2020 [1], and North American experts have recently published neonatal recommendations for training, credentialing, and quality assurance [4]. Concerns about safety persist nonetheless. In its list of health technology hazards for 2020, the Emergency Care Research Institute (ECRI) ranked POCUS second and warned that safeguards for user training, experience, and skill had not kept pace with the speed of adoption [5]. In response to these concerns, a risk assessment framework for pediatric and neonatal POCUS was later proposed [6]. It is a useful starting point, but it was built mainly around procedural and cardiac applications and around institutional and medicolegal risk. It does not give the bedside clinician a working classification of the errors themselves.

Ultrasound is also not free of biological risk. Wider use of lung ultrasound has reduced the number of chest radiographs, and therefore radiation exposure, in ventilated and CPAP-supported neonates [7]. The thermal and mechanical effects of ultrasound still deserve attention, especially in preterm infants.

This perspective proposes a structured way of thinking about the pitfalls of non-cardiac neonatal POCUS (lung, cranial, and abdominal), with the aim of supporting safer practice, training, and quality assurance. Cardiac POCUS is left aside because it has its own extensive consensus literature and because our own experience lies mainly with non-cardiac applications. Its particular position is discussed in Section 10.

## 2. A Gap in the Literature: Under-Reporting of POCUS Errors

Despite a large body of work on diagnostic accuracy, scoring systems, and procedural guidance, the neonatal POCUS literature says relatively little about when and why the technique fails [2]. Two reviews of the limitations of neonatal lung ultrasound are informative, but they deal mainly with situations in which air reflects the entire beam, such as deep congenital pulmonary airway malformations, bullae, subcutaneous or interstitial emphysema, and estimation of pneumothorax size [8]. Errors throughout the whole examination, from the decision to scan to the final clinical decision, have received much less attention.

Radiology offers a useful contrast. The American College of Radiology’s RADPEER program, introduced in 2002, asks radiologists to re-score a sample of their colleagues’ previous interpretations and has produced continuous, quantified data on discrepancy rates [9]. Neonatal POCUS has no comparable system, and to our knowledge no systematic estimate of its error rate has been published. We read this absence as a sign of under-reporting rather than as evidence that misdiagnosis is rare.

The supporting infrastructure is also incomplete. Among academic pediatric critical care programs performing diagnostic ultrasound, fewer than one in four had the core elements of a sustainable program (image storage, documentation, quality assurance, and credentialing), and no division had credentialed more than a quarter of its clinicians [6]. Neonatologists report fear of missing a diagnosis, legal concerns, difficult coordination with subspecialists, and a lack of training, experts, equipment, time, and funding as the main barriers to implementation [2].

Litigation data are sometimes reassuring. Reviews of lawsuits involving emergency physicians, and of those involving neonatologists and pediatric subspecialists, found that the usual allegation was a failure to perform an ultrasound, or to perform it in time, rather than misinterpretation [10,11]. This shows that misinterpretation rarely reaches the courts or the journals. It does not show that it rarely happens.

## 3. A Framework of Error Domains

Several authors have grouped POCUS errors along the examination process. Brockman and Wong recently classified pitfalls in adult POCUS into image acquisition, interpretation, and clinical integration [12]. The I-AIM model (indication, acquisition, interpretation, medical decision-making), developed for teaching and performing focused sonography, adds the decision to scan as a separate step [13]. We combine the two and adapt them to the newborn, using four error domains (Table 1). Our contribution lies in the neonatal content of each domain rather than in the structure itself.

Acquisition errors in the newborn often reflect patient size and equipment choice. The open anterior fontanelle is the natural window for neurosonography, not an obstacle. Relying on it alone, however, leaves the posterior fossa poorly seen, and supplementary mastoid and posterior fontanelle windows are needed to detect lesions such as cerebellar hemorrhage [14]. Dedicated neonatal cerebral ultrasound uses high-frequency transducers, generally in the 7.5 to 18 MHz range, usually combining a small-footprint probe for the standard coronal and sagittal planes with a linear probe for the cortex, the extra-axial spaces, and the superior sagittal sinus [14]. A general-purpose abdominal curvilinear probe gives poor near-field resolution in a small head. In older infants, a narrowing fontanelle progressively limits the window.

**Figure 1:**
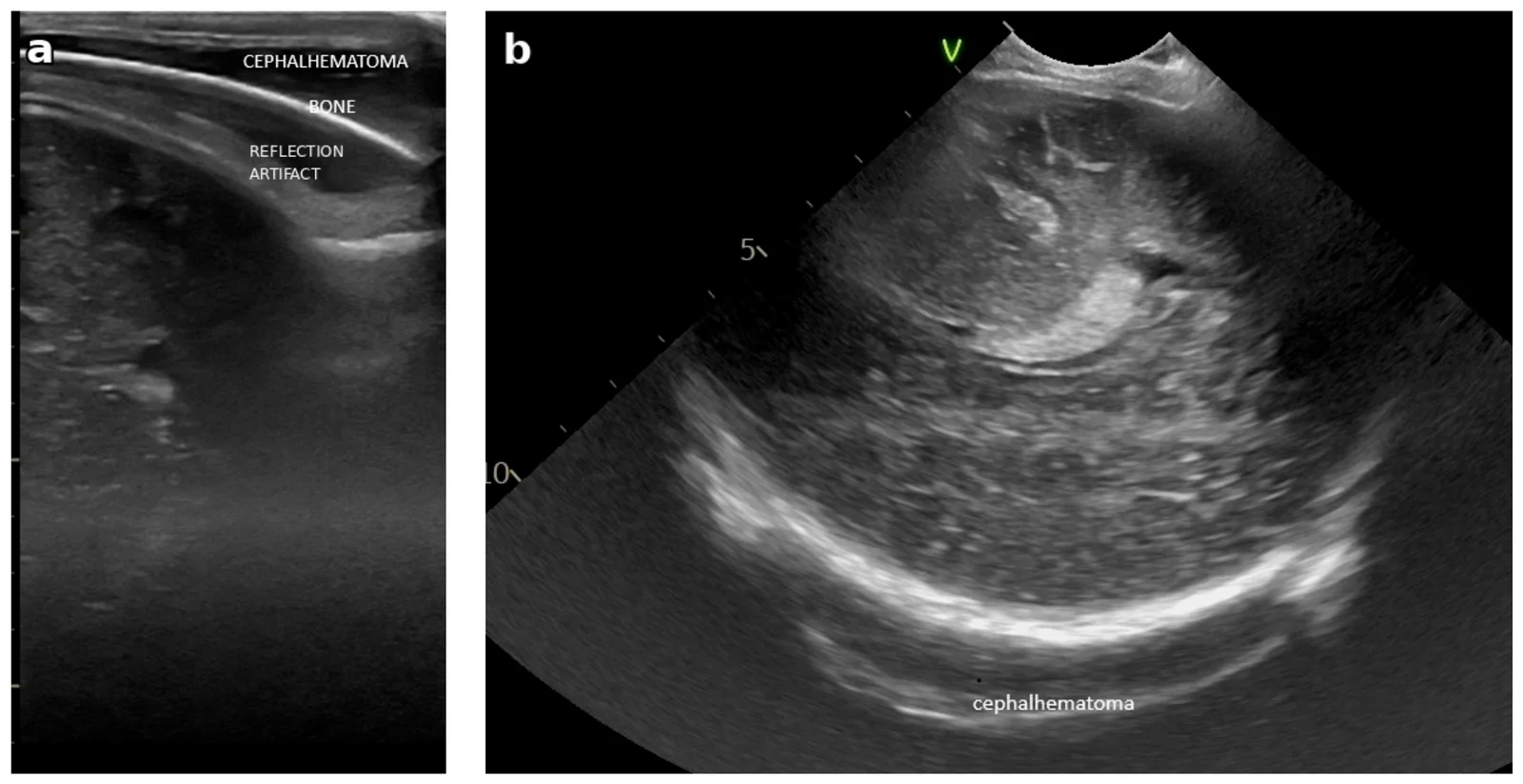
An artifact misinterpreted as pathology in a neonate with a cephalhematoma. (**a**) A bedside scan with a linear probe placed over the cephalhematoma. A reflection of the hematoma through the skull was misread as a possible epidural hematoma, and a specialist was called. (**b**) Specialist examination using a transtemporal view from the side opposite the cephalhematoma, which avoids the reflection. The brain parenchyma is intact and no epidural collection is seen.

**Figure 2:**
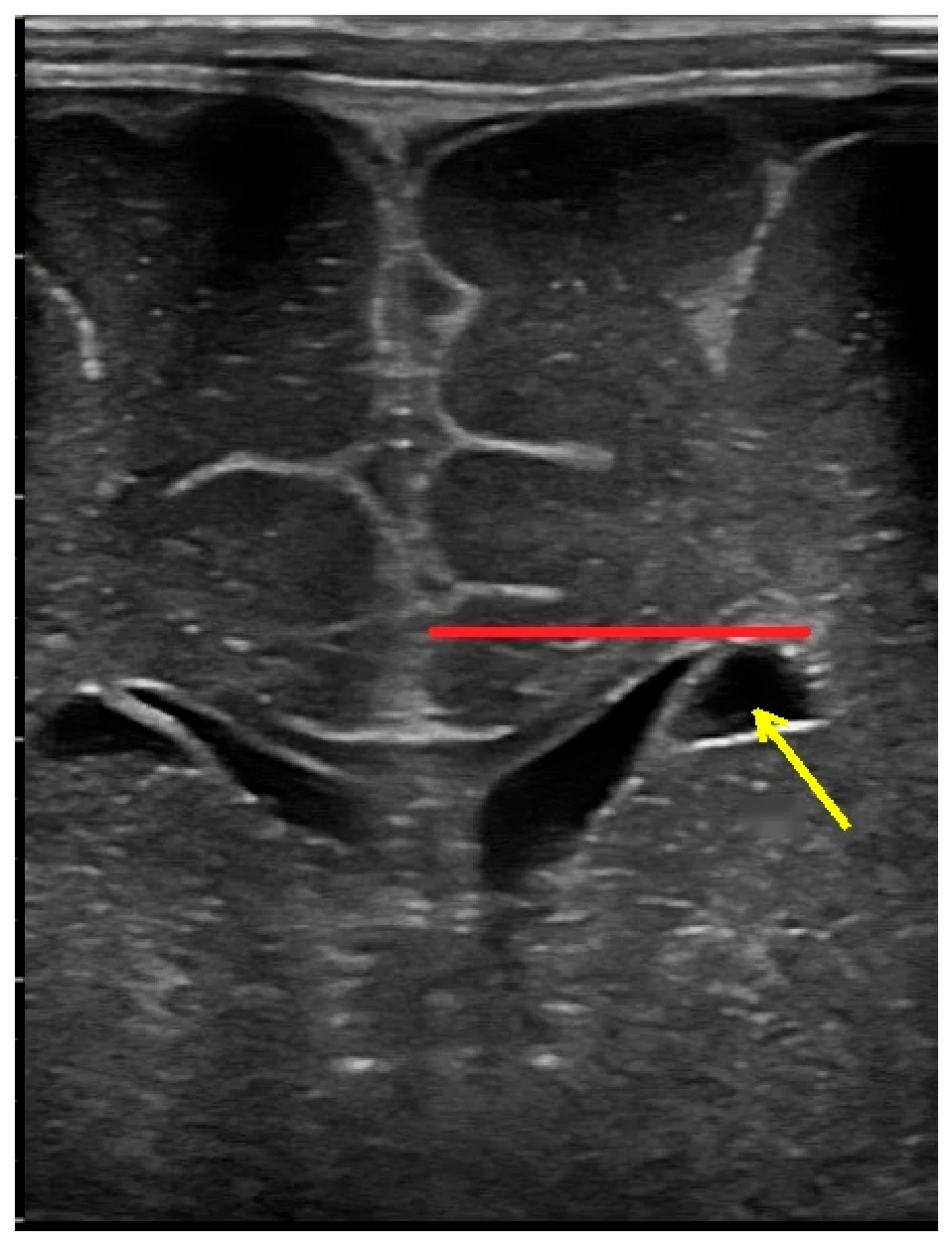
A normal variant misread as pathology. Coronal cranial ultrasound in a preterm infant with bilateral connatal (frontal horn) cysts, initially taken for periventricular white matter lesions. The arrow marks the cyst on the right-hand side of the image. A horizontal line drawn through the lateral angles of the frontal horns (red line) helps the distinction: the connatal cyst lies below this line, next to the tip of the frontal horn, whereas cystic white matter lesions usually lie above it. Connatal cysts are a benign finding in preterm infants [15].

Interpretation errors are illustrated in Figure 1 and Figure 2. In both cases a normal structure or an artifact was taken for disease, and a more experienced examiner resolved the question with a different acoustic window or a simple anatomical landmark.

**Figure 3:**
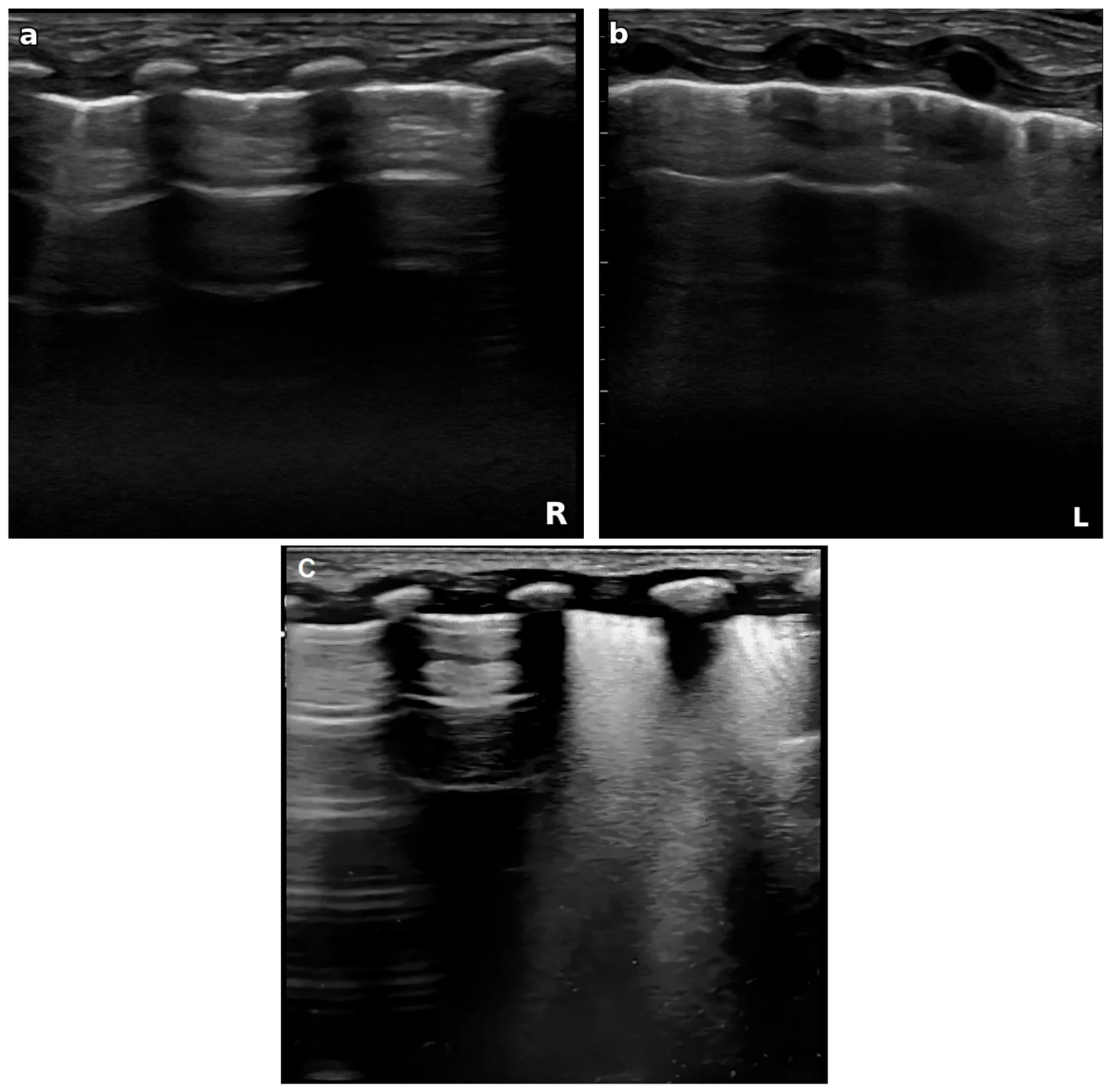
A reassuring lung scan given too much weight: an error of clinical integration. (**a**) Right lung; (**b**) left lung [scanning zone—anterior upper zone]. A late preterm infant (34 weeks) with respiratory distress was examined at 2 h of life while on CPAP. Both sides show a regular pleural line with lung sliding and A-lines, with only occasional B-lines, an appearance close to normal aeration. The scan was read as compatible with transient tachypnea of the newborn (TTN), and the infant was kept on CPAP with parenteral nutrition only. Three facts were not taken into account: maternal vaginal colonization with group B *Streptococcus*, rising hypercarbia, and worsening respiratory distress. The infant developed respiratory acidosis and progressive distress, was intubated and ventilated, and antibiotics were started. The blood culture grew group B *Streptococcus* at 72 h. Ventilation lasted 5 days and the outcome was favorable. An A-predominant pattern obtained under CPAP does not by itself establish TTN, and a near-normal scan early in life does not exclude evolving sepsis or pneumonia. The positive culture confirms the infection but says nothing about the accuracy of the scan. The error lay in letting a reassuring image outweigh the risk factors and the clinical trajectory. (**c**) A typical image for transient tachypnea, left lung, lateral scan—the primary finding is the double lung point, which is a highly specific sonographic sign for Transient Tachypnea of the Newborn; Left Side (Superior): You can observe distinct horizontal A-lines beneath the pleural line, representing a normal, well-aerated lung. Right Side (Inferior): Prominent vertical B-lines extend downward, indicating interstitial fluid or alveolar edema.

Clinical integration errors are, in our view, the most dangerous, because they follow an examination that was technically acceptable and correctly described. The case in Figure 3 shows how a near-normal lung scan can create false reassurance. Congenital diaphragmatic hernia offers a second example. Corsini et al. described the typical lung ultrasound findings as absent A-lines over the affected area, partial absence of the pleural and diaphragmatic lines, and moving bowel loops within the thorax [16]. An operator who scans only one or two anterior zones, or who is not familiar with these signs, may miss them or take the bowel into consolidation. Therefore, these findings should be read against the clinical presentation and, when available, prenatal imaging. Coexisting conditions such as pneumonia, early-onset sepsis, or air leak can also change both the appearance of the lung and its clinical meaning [3].

## 4. General Ultrasound Errors Versus POCUS-Specific Errors

Some errors can occur in any ultrasound examination. Others are specific to POCUS, which non-radiologists usually perform at the bedside, under time pressure, and without a second reader. Table 2 summarizes this distinction.

### 4.1. Physics- and Technique-Related Errors

Incorrect gain, focus, depth, or frequency and inappropriate Doppler settings are errors common to all ultrasound users. Neonatal scanning usually calls for high-frequency transducers, because the structures of interest lie only a few centimeters deep [2]. Lung ultrasound relies on artifacts generated at the pleural interface. Post-processing designed for solid organs, such as speckle reduction (“smoothing”) filters and spatial compound imaging, alters these artifacts and can distort the appearance and apparent number of B-lines. A dedicated lung preset with these functions switched off is therefore advisable [17,18]. Doppler has its own trap: a velocity scale set for adult vessels can suggest that flow is absent when it is simply too slow to register.

### 4.2. Artifacts and Anatomical Variants

Mistaking an artifact for disease is a recurring problem in every organ system. In the lung, A-lines and isolated B-lines are normal or near-normal findings, and B-lines are expected during the first day or two of life while fetal lung fluid clears [19]. Even the lung point, the most specific sign of pneumothorax, has variants such as the double lung point, septated pneumothorax, and the “hydro-point” of hydropneumothorax, which can lead an inexperienced operator to over- or under-call the diagnosis [20].

Absent lung sliding needs particular care. Sliding, B-lines, or a lung pulse rule out pneumothorax at the site examined, but absent sliding alone is not specific [21]. It is also seen with apnea, intubation of one main bronchus, pleural adhesions, extensive consolidation, and high distending pressure. In ventilated adults, lung sliding was falsely absent in 4.5% of evaluations at a positive end-expiratory pressure (PEEP) of 15 cmH_2_O and in 26.9% at 20 cmH_2_O [22]. These pressures are well above those used in neonates, so the figures cannot be transferred directly. The direction of the effect remains relevant, however, since high mean airway pressure and gas trapping may reduce sliding in ventilated newborns as well. A pneumothorax diagnosis should rest on a lung point, a consistent pattern across zones, and the clinical picture, rather than a single sign [23]. Lung ultrasound also cannot measure hyperinflation, which may mimic pneumothorax.

Neonate-specific variants add a further layer of difficulty. Ventricular asymmetry, a wide anterior fontanelle, the shape of the thymus, and the normal appearance of the preterm bowel can all be misread by an inexperienced examiner. Cranial findings also need their clinical and developmental context. Studies correlating head ultrasound at term-equivalent age with general movements assessment, and with neuromotor outcome at 12 to 24 months corrected age, show that ventricular and other cerebral measurements become meaningful when combined with functional neurological assessment rather than read in isolation [24,25].

In the abdomen, gas within the bowel lumen produces bright reverberations that can resemble the echogenic foci of pneumatosis intestinalis. Gentle probe pressure helps, because intraluminal gas moves while gas trapped in the bowel wall does not [26]. Absent color Doppler signal in the bowel wall should also be read with caution: the velocity scale must be lowered as far as possible before absent perfusion is accepted [26,27]. Peristalsis is a further trap. Normal preterm bowel may show variable and sometimes sluggish motility, while reduced or absent peristalsis is a feature of established necrotizing enterocolitis (NEC) [26]. Motility should therefore be judged together with wall thickness, echogenicity, perfusion, free fluid, and portal venous gas, never on its own.

### 4.3. Cognitive Biases and Human Factors

This is where POCUS differs most from a formal radiological examination with a second reader. Confirmation bias arises when the operator starts with a hypothesis and notices supporting findings more readily than contradicting ones. Context or expectation bias is closely related: knowing the clinical question changes how an ambiguous image is read. Time pressure adds to both. Scans are often performed during an acute event, on an infant who is moving, crying, or surrounded by tubes and lines.

Three short scenarios show how these biases look in practice. A neonatologist who suspects sepsis finds a few B-lines in a 6-h-old infant and reads them as pneumonia, discounting the more likely explanation of normal lung fluid clearance. This is confirmation bias. A preterm infant with respiratory distress syndrome (RDS) who has received surfactant suddenly desaturates. The operator, anchored on the admission diagnosis, sees posterior consolidation, attributes the deterioration to worsening RDS or atelectasis, and does not look for a lung point in the anterior zones, so a pneumothorax is missed. This is anchoring. An operator who has just diagnosed a severe intraventricular hemorrhage over-reads a normal but prominent choroid plexus in the next infant. This is availability bias, in which a recent and memorable case shapes the next interpretation.

Fatigue and uneven experience compound these effects. The pediatric POCUS risk framework lists inadequate training, inadequate supervision, poor documentation, and the lack of any longitudinal assessment of individual skill as factors that raise the risk to patients, providers, and institutions even when the equipment performs as expected [6]. The same authors point out that the severity of risk varies widely between applications. A failed ultrasound-guided peripheral cannulation carries a very different risk from a misread assessment of cardiac function in a child later found to have myocarditis [6].

### 4.4. Normal Findings and Their Look-Alikes

Teaching normal and abnormal findings side by side is one of the most direct ways to prevent interpretation errors. Table 3 summarizes the discriminating features for three pairs that are frequently confused at the bedside.

## 5. Patient Safety: Biological Effects and the ALARA Principle

Ultrasound does not use ionizing radiation, but it is not free of risk. The recognized concerns are tissue heating and mechanical effects, including inertial and non-inertial cavitation. No study has shown actual harm from diagnostic ultrasound in human neonates, but extremely preterm infants are, in theory, more vulnerable because of their immature tissues [6].

The European Federation of Societies for Ultrasound in Medicine and Biology (EFSUMB) Clinical Safety Statement, last revised in 2019, presents the mechanical index (MI) and thermal index (TI) as the safety indices displayed to the operator and confirms that the ALARA principle (as low as reasonably achievable) should guide every diagnostic examination [28]. The British Medical Ultrasound Society gives more concrete advice for neonatal scanning. The TI and MI should be displayed whenever either exceeds 0.4. Exposure time of the neonatal central nervous system should be restricted once TI exceeds 0.7, and that of other neonatal structures once TI exceeds 1.0. An MI above 0.3 carries a theoretical possibility of minor damage to the neonatal lung or intestine and should prompt a shorter exposure if that setting is clinically necessary [29]. Published neonatal lung ultrasound guidelines accordingly recommend a preset that keeps the MI at or below 0.3, since B-mode lung imaging gains nothing from higher output [17]. Table 4 summarizes the main concerns by tissue.

Doppler delivers considerably more acoustic energy than B-mode imaging. It should be kept short and, in the most immature infants, avoided when not strictly needed. We also propose, as our own suggestion rather than an established standard, that the peak MI and TI reached during a neonatal POCUS examination be recorded in the report. This would parallel the recording of dose in radiology, create an auditable record, and reinforce the ALARA habit at the bedside.

## 6. Infection Control and Equipment Safety

Probes and gel shared between patients can carry pathogens, and preterm infants are among the most vulnerable patients in any hospital. Probes can become contaminated with body fluids and act as vectors for cross-transmission [6]. The European Society of Radiology recommends cleaning and disinfection of the transducer before first use and after every examination. Low-level disinfection is considered sufficient for standard examinations on intact skin, whereas all interventions require high-level disinfection, a dedicated transducer cover, and sterile gel [31].

These rules need some adaptation in the NICU. The skin of extremely preterm infants is thin and easily damaged, and many scans are performed close to umbilical catheters, central lines, or surgical wounds. In these situations, we consider it prudent to treat the examination as contact with non-intact skin and to apply the higher standard, using disinfectants compatible with the manufacturer’s instructions. Gel deserves the same attention. Contaminated gel, including unopened non-sterile product, has been linked to outbreaks of *Burkholderia cepacia* complex bacteremia in neonatal and adult intensive care units [32]. Single-use sterile gel sachets are a simple safeguard. The keyboard, cable, and trolley should be cleaned between patients, and a machine should not move from an isolation room to other incubators without cleaning. Recording probe cleaning in the examination report closes the loop.

Equipment also ages. Transducer elements can fail and processing speed can fall over the working life of a machine, gradually degrading image quality [6]. Periodic technical checks of probes and presets belong in the quality assurance program described below.

## 7. Training, Credentialing, and Quality Assurance

### 7.1. Where the Field Stands

At present, no national or international body certifies neonatal POCUS operators or defines who may perform which examination. The authority to scan rests with individual institutions, through local credentialing and the granting of privileges [4]. Recent guidance from the National Neonatal POCUS Collaborative is the most detailed proposal so far [4]. It separates basic from advanced applications and suggests 25 to 50 studies per diagnostic application, with a mix of normal and abnormal examinations, excluding cardiac POCUS. The authors stress that these numbers are expert recommendations rather than strict requirements, and they favor competency-based over time- or number-based assessment [4]. The pathway we propose below is consistent with this guidance and is intended to be adapted locally.

### 7.2. A Stepwise Pathway

**Figure 4:**
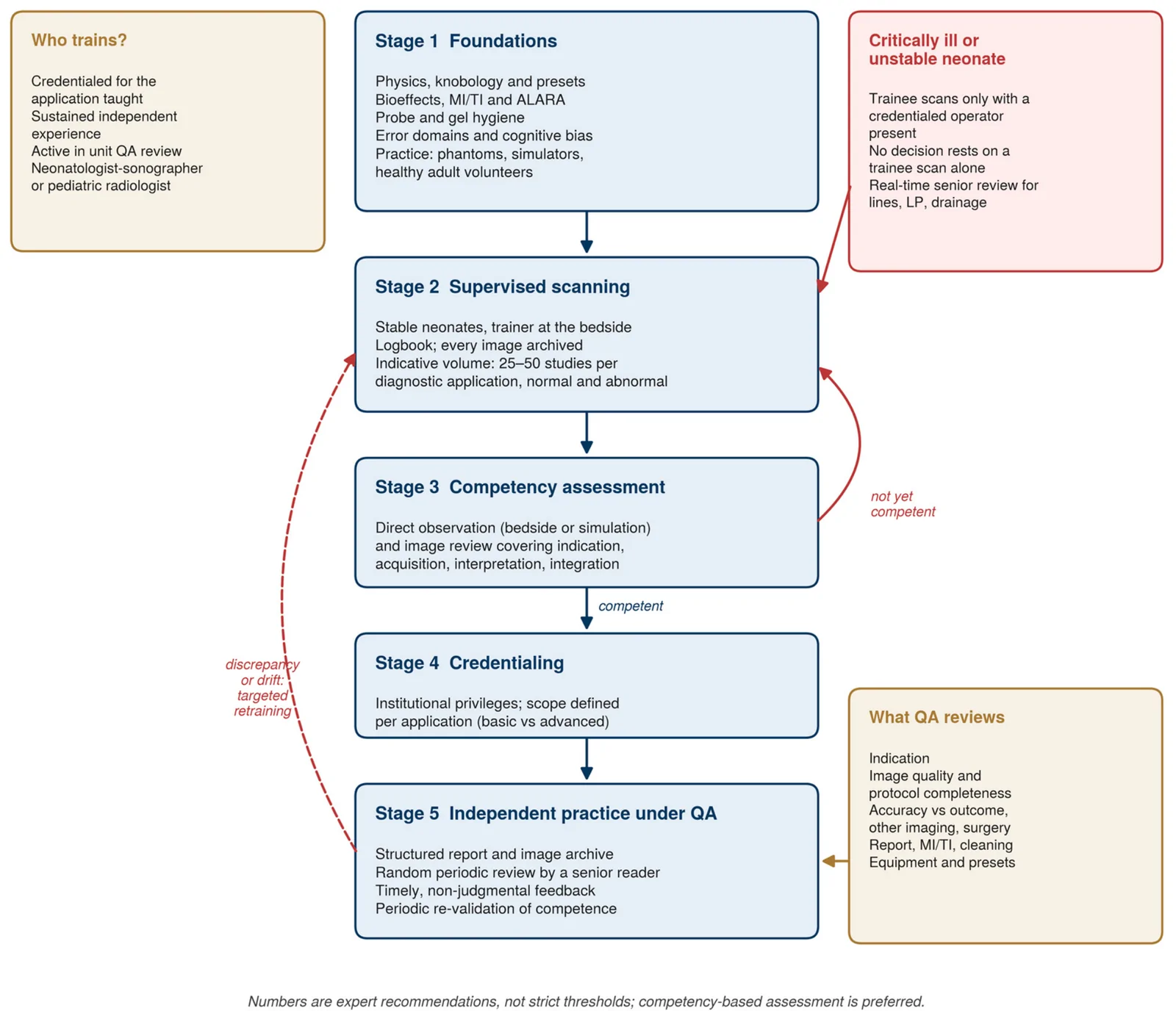
The proposed pathway for training, credentialing, and quality assurance in non-cardiac neonatal POCUS, informed by recent expert guidance [4]. Scan numbers are indicative. Competency-based assessment is preferred to fixed thresholds. LP, lumbar puncture; MI, mechanical index; QA, quality assurance; TI, thermal index.

Figure 4 outlines the pathway. Training begins with theory and simulation: ultrasound physics, machine controls and presets, bioeffects and ALARA, probe and gel hygiene, and the error domains and cognitive biases described above. Basic probe handling and machine operation can be practiced on phantoms, simulators, and healthy adult volunteers, where scanning time carries no clinical cost. Neonatal patterns, however, can only be learned on neonates.

Supervised scanning then begins on stable infants, with the trainer at the bedside, a logbook, and archiving of every image for review. Competence is assessed by direct observation, at the bedside or in simulation, together with review of archived images, and it should cover all four domains: indication, acquisition, interpretation, and integration. A trainee who is not yet competent returns to supervised practice. Credentialing follows, with privileges defined application by application.

Critically ill or unstable neonates need a stricter rule. Until competence in the relevant application is documented, a trainee should scan such infants only with a credentialed operator present, and no management decision should rest on a trainee’s scan alone. Procedures such as central line positioning, lumbar puncture, or drainage of an effusion benefit from real-time senior review [2].

Supervision should be provided by trainers who are credentialed for the application they teach, have sustained independent experience with it, and take part in the unit’s quality review. In practice, this means an experienced neonatologist–sonographer or a pediatric radiologist, and a pediatric cardiologist or TnECHO-trained neonatologist for cardiac work. Experience as an instructor in a recognized course is an advantage.

### 7.3. Quality Assurance

Quality assurance should cover the indication, image quality and protocol completeness, the accuracy of interpretation, and documentation. Fraga et al. recommend archiving all POCUS images with the report, auditing interpretations against outcomes, other tests, and surgical or pathological findings, and giving timely, non-judgmental feedback. Until a unit has its own quality team, they advise auditing every image, with help from radiology, cardiology, and local experts [4]. Once a program matures, a RADPEER-like approach becomes feasible: a fixed random sample of studies is re-scored by a senior reader each quarter, giving the unit its own discrepancy rate over time and helping to detect technical drift [9]. Asynchronous review suits most trainee scans, but some situations call for real-time feedback because a decision is being made at that moment [2]. Quality assurance also covers equipment: probe integrity, preset configuration, and cleaning records. Discrepancies should lead to targeted retraining rather than blame.

## 8. Further Safeguards

### 8.1. Standardized Protocols and Checklists

Written protocols and checklists for each type of neonatal examination reduce errors of omission and make the examination less dependent on individual experience. The SAFE protocol (Sonographic Algorithm for liFe threatening Emergencies) and its successor SAFE-R apply this approach to the highest-acuity setting. They offer a simple, ordered rule-in and rule-out sequence covering cardiac tamponade, pneumothorax, pleural effusion, and myocardial function, designed for the average neonatologist with limited additional training [2,3,33].

### 8.2. Debiasing Strategies

Debiasing belongs at the interpretive stage of every examination. Two questions asked before an interpretation is finalized, “what else could this be?” and “what would I conclude if I knew nothing about this patient?”, directly counter confirmation and context bias. They also fit the general rule that clinical and laboratory data should be integrated with the image rather than replaced by it [3]. A related cognitive-forcing strategy, the “STOP” mnemonic (stop, think, observe, then proceed), builds a short pause into the workflow before a finding is acted upon. In our own practice, we also try to prove the first hypothesis wrong before accepting it.

### 8.3. Interdisciplinary Collaboration

Collaboration with radiology remains essential. POCUS does not replace a comprehensive ultrasound examination, and a specialist opinion should be sought when a finding is equivocal or when the clinical picture does not match the image. Reviews of the limitations of neonatal lung ultrasound, for example, recommend chest radiography or computed tomography to evaluate bullae, subcutaneous and interstitial emphysema, and the size of a pneumothorax, because air makes these entities partly or wholly invisible to ultrasound [8]. A regular joint case review between neonatal POCUS operators and radiology or cardiology colleagues is a simple, low-cost step. More broadly, ultrasound works best as an extension of the physical examination that answers a focused clinical question, not as a substitute for clinical skill. Used this way, adult studies show it improves diagnostic accuracy beyond physical examination alone and reduces unnecessary downstream testing [34].

### 8.4. Documentation and Structured Reporting

Documentation and image storage are central to safety and remain underrepresented in neonatal POCUS. A truly structured report uses a predefined, IT-supported format and standardized terminology rather than free-text dictation. This improves the consistency and reproducibility of any imaging report and makes later data analysis possible. The distinction between standardization (agreement on content and vocabulary) and structured reporting (the tool that produces the report) is directly relevant to the design of neonatal POCUS templates [35]. Neonatal lung ultrasound now has specific proposals: recent standard-setting work defines a common terminology and examination approach [18], and the Pleura-ABCDE protocol offers a region-by-region scheme for documenting the pleura, A-lines, B-lines, consolidation, dynamic signs, and effusion [36]. At a minimum, each neonatal POCUS report should state the indication, the operator, the probe and preset, the zones or windows examined, the findings and their interpretation, any limitations, and the archiving reference. Probe cleaning and, as proposed above, the peak MI and TI can be added. The key takeaways from this review are summarized in Box 1.

Box 1 **Key messages.**

- The absence of published data on neonatal POCUS errors is not evidence that errors are rare. Errors can be grouped into four domains, and failures of clinical integration are the most dangerous because they follow a technically adequate scan.
- Bedside cognitive bias, look-alike findings, and gaps in output control and probe hygiene are largely preventable with structured training, checklists, and simple debiasing habits.
- Competency-based credentialing, image archiving with regular expert review, and structured reporting should be seen as part of a POCUS program, not as optional additions.

## 9. A Note on Value: Safety Investment Is Not Simply a Cost

Cost is a frequent objection to formal POCUS training, credentialing, and quality infrastructure. The available economic evidence, drawn largely from adult internal medicine, points the other way. A prospective cost–benefit analysis of bedside ultrasound in an internal medicine department found a break-even point of 734 examinations, corresponding to about €82,000 and reached within 406 days, after which the program produced consistent savings through more appropriate diagnosis-related group coding, shorter hospital stays, and less downstream imaging [37]. This analysis was not performed in neonates. It does suggest, however, that the investment needed for training, equipment, and quality assurance, the very infrastructure this article argues for, can be recovered through better diagnostic efficiency.

In the neonatal setting, the economic argument is strengthened by the potential of lung ultrasound to reduce chest radiographs, with their radiation exposure and handling of fragile infants, as shown in a quality management project in ventilated and CPAP-supported neonates [7]. The same logic plausibly extends to fewer radiographs for confirming central line position when position is checked by ultrasound, and occasionally to avoiding head computed tomography. Whether structured POCUS also shortens length of stay through earlier diagnosis remains a hypothesis. Dedicated neonatal cost–benefit studies are needed before firmer conclusions can be drawn.

## 10. Limitations

This perspective is limited to lung, cranial, and abdominal POCUS. Cardiac ultrasound in the NICU nevertheless deserves more than a passing mention, and the distinction between cardiac POCUS and targeted neonatal echocardiography (TnECHO) matters for safety. TnECHO is a comprehensive, quantitative hemodynamic assessment performed by neonatologists with advanced training, ordinarily after an initial comprehensive echocardiogram has excluded congenital heart disease. It guides decisions about the ductus arteriosus, pulmonary hypertension, and cardiovascular therapy, and it has its own training standards and quality requirements [38]. The distinction does not mean that all cardiac ultrasound should be left to TnECHO specialists. With structured training, a neonatologist can reasonably acquire basic, routinely useful skills: qualitative assessment of systolic function and cardiac filling, detection of pericardial effusion, recognition of a patent ductus arteriosus with shunt direction, and signs suggesting pulmonary hypertension [38,39]. Recent recommendations list qualitative cardiac function, filling, and pericardial effusion among the basic neonatal POCUS applications [4]. Quantitative measures such as ejection fraction and cardiac output can be learned with further training, but full hemodynamic interpretation and, above all, the exclusion of structural heart disease require TnECHO or pediatric cardiology support. The errors specific to cardiac imaging deserve a separate analysis, which we have not attempted here.

The four-domain framework is a conceptual synthesis, not a validated instrument, and we have not tested it for inter-rater reliability or its ability to reduce errors in practice. As noted in Section 2, the evidence on POCUS errors is sparse, and several observations discussed here are extrapolated from adult critical care and general pediatric literature rather than from neonatal error-reporting studies. The pathway in Figure 4 reflects expert opinion and our own experience and should be adapted to local resources.

Two further limitations deserve mention. First, artificial intelligence is increasingly being built into image acquisition and interpretation. It may reduce some acquisition and interpretation errors, but it will also introduce new, automation-related errors that this framework does not yet address. Second, our proposals assume a level of institutional resources and subspecialty support that is not available everywhere. Units with limited access to radiology or cardiology will need to adapt them.

## 11. Conclusions

Neonatal POCUS is best regarded not as a broad screening tool but as a focused, question-driven examination performed by well-trained and appropriately supervised clinicians. Making sound, rapid decisions under clinical pressure is much harder without solid grounding in both the technique and its limits. POCUS is a skill. It requires structured training, disciplined technique, awareness of one’s own cognitive biases, and a willingness to ask for a second opinion when the image and the clinical picture disagree. A framework that separates errors of indication, acquisition, interpretation, and clinical integration, combined with attention to the human factors of bedside imaging, offers a practical starting point for the training curricula, quality assurance programs, and structured reporting systems that non-cardiac neonatal POCUS still largely lacks.

## Figures and Tables

**Table 1:** Error domains in non-cardiac neonatal POCUS, adapted from the I-AIM model [13] and from Brockman and Wong [12].

Error Domain	What Goes Wrong	Neonatal Examples
1. Indication	The scan is performed without a clear question, for a question POCUS cannot answer, or not when it should be.	Using lung ultrasound to exclude a lesion that does not reach the pleura, such as a deep congenital pulmonary airway malformation; relying on a limited cranial scan when full neurosonography is needed; not scanning an infant who deteriorates suddenly on the ventilator.
2. Image acquisition	Technique, machine settings, probe choice, or the acoustic window limit the image.	Smoothing or compound imaging left on during lung imaging; a Doppler velocity scale set too high for low-velocity neonatal flow; cranial scanning with an unsuitable probe, or through the anterior fontanelle alone so that the posterior fossa is not assessed; incomplete scanning of both hemithoraces.
3. Image interpretation	A normal structure, variant, or artifact is read as disease, or the reverse.	Physiological B-lines in the first hours of life read as interstitial syndrome; normal periventricular echogenicity read as early leukomalacia; a bulky choroid plexus read as hemorrhage; intraluminal gas read as pneumatosis intestinalis (Figure 1 and Figure 2).
4. Clinical integration	A technically adequate, correctly described scan is given the wrong clinical weight.	A near-normal lung scan taken as reassurance in an infant with risk factors for sepsis (Figure 3); absent lung sliding taken as proof of pneumothorax without other signs or clinical correlation.

**Table 2:** General ultrasound pitfalls versus pitfalls specific to point-of-care use in the neonate.

Category	General Ultrasound Pitfalls	POCUS-Specific Pitfalls in the Neonate
Physics and technique	Incorrect gain, focus, depth, frequency, or Doppler settings.	The same errors, compounded by time pressure and less formal training.
Artifacts	Reverberation, acoustic shadowing, and mirror-image artifacts.	The same artifacts, interpreted without radiological back-up.
Anatomical variants	Normal variants misread as pathology.	Neonate-specific variants: ventricular asymmetry, thymic shape, connatal cysts, the appearance of the preterm bowel, and immature gyration.
Cognitive factors	Present but less studied.	Confirmation bias, anchoring on the clinical question, expectation bias, time pressure, and fatigue.
Acoustic window	Limited by body habitus.	A small or closing fontanelle in older infants; aerated lung; tubes, lines, chest drains, dressings, and monitoring leads that restrict probe placement; movement and crying.

**Table 3:** Normal findings and look-alike pathology: discriminating features for teaching.

Pair	Normal or Benign Finding	Pathological Finding	Practical Pitfalls
Normal lung vs. pneumothorax [21,23]	Lung sliding in B-mode; “seashore” pattern in M-mode (granular texture below the pleural line); B-lines or lung pulse may be present.	No sliding; “stratosphere” (barcode) pattern in M-mode; no B-lines; lung point, where sliding and non-sliding lung meet, is the specific sign.	Absent sliding alone is not specific (apnea, one-sided intubation, adhesions, consolidation, high airway pressure). Scan the non-dependent anterior chest in the supine infant.
Normal ventricles and choroid plexus vs. germinal matrix or intraventricular hemorrhage [14]	Mild ventricular asymmetry without clot or abnormal echogenicity; smooth choroid plexus that tapers anteriorly and does not extend in front of the caudothalamic groove.	Echogenic focus at the caudothalamic groove, in front of the choroid plexus; intraventricular clot; irregular or bulky plexus; ventricular dilatation.	Confirm in both coronal and parasagittal planes. Use the mastoid window for the posterior fossa. Compare with previous scans.
Normal bowel vs. NEC [26,27]	Wall thickness about 1.1–2.6 mm with preserved layers; visible peristalsis; mural color Doppler signal present; gas moving within the lumen.	Thickened or thinned wall with loss of layers; pneumatosis (fixed echogenic foci within the wall); portal venous gas; reduced or absent peristalsis; increased or absent mural flow; complex free fluid.	Intraluminal gas can mimic pneumatosis (test with probe pressure). Lower the Doppler scale before calling flow absent. Peristalsis alone is not diagnostic.

**Table 4:** Thermal and mechanical bioeffects of diagnostic ultrasound by tissue, with practical guidance for neonatal POCUS. No harmful effect has been demonstrated in human neonates at diagnostic output; the table reflects theoretical and experimental concerns [28,29].

Tissue	Main Concern	Why It Matters in the Neonate	Practical Guidance
Eye	Thermal	The lens and vitreous have little or no perfusion, so heat is poorly dissipated. The eye is regarded as the tissue most sensitive to heating.	Avoid directing the beam through the orbit. If unavoidable, use an ophthalmic preset; regulatory limits for ophthalmic use are TI ≤ 1.0, MI ≤ 0.23, and I_SPTA.3_ ≤ 50 mW/cm^2^ [30].
Brain	Thermal	Heating is greatest where bone lies in the beam; as the skull ossifies, bone increasingly lies in the beam path.	Restrict exposure time when TI exceeds 0.7 [29]. Keep spectral Doppler of cerebral vessels brief. Freeze the image when not actively scanning.
Lung	Mechanical	Gas–tissue interfaces favor mechanical effects; alveolar capillary hemorrhage has been produced in animal models at diagnostic outputs.	Use a lung preset with MI ≤ 0.3 [17,29]. B-mode lung imaging gains nothing from higher output.
Bowel	Mechanical	Intraluminal gas bodies can act as cavitation nuclei; petechial hemorrhage of the intestinal wall has been described in animal studies.	Keep MI ≤ 0.3 when possible; if a higher setting is needed, shorten exposure [29].
Vessels	Thermal	Pulsed-wave and color Doppler deliver the highest time-averaged intensities, especially when bone lies near the focal zone.	Use the smallest color box and sample volume, the lowest effective output, and the shortest dwell time.
Heart	Thermal	Doppler interrogation during functional assessment can be prolonged; the thin neonatal chest wall places the heart close to the transducer.	Limit Doppler dwell time and restrict exposure when TI exceeds 1.0 [29].

I_SPTA.3_, derated spatial-peak temporal-average intensity; MI, mechanical index; TI, thermal index.

## Data Availability

No new data were created or analyzed in this study. Data sharing is not applicable to this article.
